# Identification of a Novel Allele of *TaCKX6a02* Associated with Grain Size, Filling Rate and Weight of Common Wheat

**DOI:** 10.1371/journal.pone.0144765

**Published:** 2015-12-14

**Authors:** Jie Lu, Cheng Chang, Hai-Ping Zhang, Sheng-Xing Wang, Genlou Sun, Shi-He Xiao, Chuan-Xi Ma

**Affiliations:** 1 College of Agronomy, Anhui Agricultural University, Key Laboratory of Wheat Biology and Genetic Improvement on Southern Yellow & Huai River Valley, the Ministry of Agriculture, Hefei, 230036, China; 2 Institute of Crop Sciences, National Wheat Improvement Centre/The National Key Facility for Crop Gene Resources and Genetic Improvement, Chinese Academy of Agricultural Sciences (CAAS), Beijing, 100081, China; 3 Department of Biology, Saint Mary’s University, Halifax, NS, B3H3C3, Canada; Institute of Genetics and Developmental Biology, CHINA

## Abstract

Cytokinin oxidase (CKX) plays a crucial role in plant growth and development by reversibly inactivating cytokinin (CTK). Twenty-four primer pairs, designed from ESTs of the *TaCKX* genes family of common wheat, were used to identify their allelic variations associated with grain size, weight, and filling rate in 169 recombinant inbred lines (RIL) derived from Jing 411 × Hongmangchun 21. *TaCKX6a02*, a member of *TaCKX* gene family, amplified by primer pair *T31–32*, showed a close association with grain traits in this RIL population. Statistical analysis indicated that allelic variation of *TaCKX6a02* had significant correlation with grain size, weight, and filling rate (GFR; *P* < 0.001) under varied environments. The *TaCKX6a02-D1a* allele from Jing411 significantly increased grain size, weight and grain filling rate, compared with *TaCKX6a02-D1b* from Hongmangchun 21. *TaCKX6a02* was located on chromosome 3DS in the interval of *Xbarc1119* and *Xbarc1162*, with a genetic distance of 1.4 cM. The location was further confirmed using Chinese Spring nulli–tetrasomic lines. A major QTL (quantitative trait locus) tightly linked to *TaCKX6a02* was detected in the RIL population, explaining 17.1~38.2% of phenotype variations for grain size, weight, GFRmax and GFRmean in different environments. In addition, significant effects of variations of *TaCKX6a02* on grain weight and GFR were further validated by association analysis among 102 wheat varieties in two cropping seasons. 12.8~35.1% of phenotypic variations were estimated for these genotypes. A novel 29-bp InDel behind the stop codon was detected by DNA sequence analysis between the two alleles of *TaCKX6a02-D1*. The gene-specific marker, *TKX3D*, was designed according to the novel variation, and can be used in marker-assisted selection (MAS) for grain size, weight, and GFR in common wheat.

## Introduction

As one of the key phytohormones synthesized in the root, cytokinin (CTK) regulates many important plant processes by controlling cell division and tissue differentiation [1–9]. Many researches indicate that CTK plays an important regulatory role in crop yield and related traits. Firstly, cytokinin is one key hormone in controlling grain size and weight by regulating endosperm cell numbers of crops [10–14]. Secondly, CTK also can enhance grain weight by regulating grain filling patterns of crops [15–18]. The endogenous CTK (Z+ZR) content of grain at the early grain filling stage has a close relationship with thousand-grain-weight (TGW) via regulation of starch synthase (SSase) and starch branching enzyme (Q-enzyme) activities in rice [19]. CTK also can maintain chloroplast stability of flag leaf after anthesis, significantly improving grain filling and weight [20, 21]. For other yield-related traits, such as effective tiller emergence, spikelet and floret aspects, and fertile floret and kernel numbers, CTK is generally regarded as a positive regulator [22–25].

The phytohormone, CTK, is under strict control mainly regulated by cytokinin oxidase (CKX). The CKX is considered to be only known phytohormone enzyme that can inactivate CTK irreversibly by cleaving its N^6^-side chain in a single enzymatic step [26]. Hence, cytokinin oxidase (*CKX*) genes have been proven to have a close relationship with crop yield through regulation of endogenous CTK. Ashikari et al. isolated the rice *CKX* gene (*OsCKX2*) and confirmed that reduced expression caused CTK accumulation in inflorescence meristems and increased the number of reproductive organs, resulting in enhanced grain yield [27]. As graminaceous crops, wheat, rice, barley (*Hordeum vulgare* L.), maize (*Zea mays*), and *Sorghum* spp. show large similarity and co-linearity between their genomes; therefore, wheat *CKX* genes are also involved in the regulation of grain yield. Zalewski et al. confirmed that seed numbers per plant and seed weight are improved by silencing the *HvCKX1* gene in barley and the *TaCKX1* gene in wheat [28]. Two putative *CKX* genes of wheat (*TaCKX2*.*1* and *TaCKX2*.*2*) have been cloned and are presumed to relate to grain number per spike [29]. *TaCKX6* is confirmed to have a significant association with the grain weight of common wheat [30]. Based on sequences analysis of ESTs available from the NCBI (www.ncbi.nlm.nih.gov), wheat *CKX* genes are proved to belong to a large gene-family containing at least 13 members [31]. So far, seven wheat *CKX* genes have been isolated: *TaCKX1* on chromosome 3A [32], *TaCKX2* on 7A or 7B [33], and *TaCKX2*.*1*, *TaCKX2*.*2* [29], *TaCKX3* [34], *TaCKX5* [35], and *TaCKX6* [30] on 3DS. However, little research was carried out to investigate the effects of allelic variations of *TaCKX* genes on grain filling, size, and grain weight of wheat simultaneously. The objectives of this study are to: (1) identify allelic variations of *CKX* genes for grain filling, grain size, and grain weight based on all *TaCKX* ESTs, mRNA, and DNA sequences available in the NCBI; and (2) develop a gene-specific marker for *TaCKX6a02* and validate its association with grain traits.

## Materials and Methods

### Plant Materials

One hundred and sixty-nine recombinant inbred lines (RILs) were developed from a cross between Jing 411 and Hongmangchun 21 by single-seed descent (F_2:8_ generation). Jing 411 is a high-yield winter wheat variety with a larger grain size; its TGW averaged 47.6 g over five cropping seasons (2006~2007, 2007~2008, 2008~2009, 2009~2010 and 2010~2011). Hongmangchun 21 is a Chinese low-yield landrace, with an average TGW of 19.7 g across the five cropping seasons (Table 1). The two parents show large differences in yield-related traits, including grain size, grain weight, and grain filling (Table 1). One hundred and two wheat varieties from different wheat production regions of China showing large variation in grain filling, size, and weight were used for validation of the gene marker (Table 1).

**Table 1:** Grain traits of the two parents, RIL and natural population.

Traits	Parents		RIL population (n = 169)		Natural population (n = 102)	
	Jing411	Hongmangchun21	Mean	C.V.%	Mean	C.V.%
GL (mm)	6.9	5.11	6.30(4.97–7.12)	14.15	6.33(5.01–7.11)	14.26
GW (mm)	3.82	2.4	3.05(2.33–3.94)	12.86	3.32(2.64–3.83)	10.26
GT (mm)	3.81	2.41	2.97(2.39–3.88)	11.13	3.17(2.56–3.78)	10.01
TGW(g)	47.6	19.7	36.15(18.6–53.2)	28.12	44.2(19.7–61.2)	26.38
GFR_max_	3.45	1.88	2.49(1.01–3.56)	31.04	3.08(2.13–3.51)	15.79
GFR_mean_	2.21	1.04	1.72(0.97–2.37)	22.11	2.03(1.12–2.33)	20.85

The means performance of grain traits were measured in five cropping seasons (RIL population), and two cropping seasons (natural population), respectively. The data in parentheses mean the range of grain traits values. GL, grain length; GW, grain width; GT, grain thickness; TGW, 1000-grain-weight; GFR_max_ and GFR_mean_, maximum and mean grain filling rate, respectively; RIL, recombinant inbred line.

### Field trials

The RILs and their parents were grown in randomized complete blocks, with two replicates at the Changping Experimental Station of Chinese Academy of Agricultural Sciences (Beijing: 39°54'N, 116°28'E) in the 2006~2007 and 2007~2008 cropping seasons, and the Experimental Station of Anhui Agricultural University (Hefei; 31°58'N, 117°24'E) during the 2008~2009, 2009~2010 and 2010~2011 cropping seasons. The 102 wheat varieties were also grown in randomized complete blocks with two replicates during the 2011~2012 and 2012~2013 cropping seasons at the Experimental Station of Anhui Agricultural University.

Each plot contained three 2.0-m rows spaced 25 cm apart, with 40 plants in each row. Field management followed common practices for wheat production, and the flowering time was recorded for each line.

### Recording of data for grain size, weight, and grain filling rate

Grain size and weight were determined in all seasons and locations. TGW was evaluated by weighing two samples of 1000 kernels from each genotype. Grain size, including length and width, was recorded after harvesting using the method described by Sun et al. [36], with minor modifications. Two sets of 50 grains, from each variety or RIL, were lined up length-wise along a ruler with a precision of 0.1 mm, and the average length of the samples defined as grain length (GL). The average width of the two sets of grains laid breadth-wise was defined as grain width (GW). The thickness (GT) of two sets of 20 grains was measured using vernier calipers (Kraftwelle, China) with a precision of 0.01 mm, using the method described by Dholakia et al. [37].

GFR_max_ and GFR_mean_ were determined for the RILs during the 2009~2010 and 2010~2011 cropping seasons, and 102 wheat varieties during the 2011~2012 and 2012~2013 cropping seasons at the Experimental Station of Anhui Agricultural University respectively, following the method described by Wang et al. (2008) [38]. Seven tagged spikes from each line were sampled at 5-day intervals from anthesis to maturity. The grains were then separated from the glumes, kept at 105°C for 10 min, and then at 70°C until reaching a constant weight. At this stage, the total number of grains was counted, and the weight recorded. Grain filling rates were calculated as described by Wang et al. [38].

### Genomic DNA extraction and PCR amplification

Genomic DNA was extracted from two kernels per line in accordance with Kang et al. [39]. PCR reactions were conducted using a TC412 Thermocycler (Barloworld Scientific, UK). Twenty-four primer pairs (S1 Table) were designed to characterize allelic variations of *TaCKX* genes in wheat using DNAMAN (Version 6.0) software (Lynnon Biosoft, San Ramon, CA, USA) based on EST and mRNA sequences available from the NCBI.

The PCR profile was as follows: denaturation at 94°C for 5 min; followed by 40 cycles of denaturation at 95°C for 1 min, annealing at 50~60°C for 1 min 20 s, and extension at 72°C for 2 min; with a final extension for 8 min at 72°C. The annealing temperature was modulated based on the primer pair. Each 15-μl PCR reaction contained 40 ng of genomic DNA, 10 pmol of each primer, 200 mM dNTPs, 1×PCR buffer, and 1 U *Taq* DNA polymerase (Shanghai Sangon Biological Engineering Technology & Services, Shanghai, China). PCR products were separated by 7% PAGE containing 4 M Urea.

### Sequencing of PCR fragments and statistical analysis

Purification and sequencing of PCR products, from two independent samples per wheat genotype was performed by Shanghai Sangon Biological Engineering Technology & Services. Pfu *Taq* DNA polymerase (Shanghai Sangon Biological Engineering Technology & Services, Shanghai, China) with high fidelity was used to avoid false priming; all *TaCKX* alleles were sequenced from both strands. Sequence alignment and characterization were completed using DNAMAN software. Data analysis of grain traits was conducted using SPSS software version 13.0 (IBM Software Group, Armonk, NY, USA).

### Chromosomes assignment of *TaCKX* gene

A set of Chinese Spring nulli–tetrasomic lines was used to determine the location of *TaCKX* gene. PCR amplification and separation of products was as described above.

### SSR marker screening and QTL analysis for grain weight and grain filling

Simple sequence repeat (SSR) markers, including the BARC, GWM, WMC, CFA, and CFD series were used to screen the two parents, and two bulks containing five high-grain-weight lines and five low-grain-weight lines, respectively [40]. Candidate polymorphic markers between the bulks were analyzed further in a subset of 40 RILs, including 20 lines with high-grain-weight and 20 with low-grain-weight, to confirm the polymorphism. The polymorphic markers were then used for genotyping the entire RIL population. The linkage map was constructed using Map Manager QTXb20 Version 3.0 [41]. Recombination fractions were converted into centimorgans (cM) using the Kosambi function [42]. Composite interval mapping (CIM) was performed for QTL analysis using Windows Cartographer 2.5 software (http://statgen.ncsu.edu/qtlcart/WQTLCart.htm) in accordance with the methods described by Zeng [43]. A QTL was declared when the LOD score was > 2.5 in at least two cropping seasons, and calculated from 2,000 permutations at a probability of 0.01.

### Analysis of correlation between *TaCKX* alleles and grain traits

When analyzing the correlation between *TaCKX* alleles and grain traits in the RILs population and wheat varieties, the Jing 411 allele was scored as “1” and the Hongmangchun 21 allele as “0”. Spearman rank correlation analysis and t-test were performed to test the association significance between allelic variation and grain traits, as described in previous studies [44–46]. The effect of *TaCKX* variation on the variability of grain traits was estimated by *R* ^*2*^ based on the General Linear Model [44–46]. The significance of the effect was evaluated using the GLM at 0.05, 0.01, and 0.001 levels of probability. Grain trait data analyses were conducted using SPSS software version 13.0.

## Results

### Correlation analysis between different grain traits

In this study, the grain traits of RILs such as GL, GW, GT, TGW, GFR_mean_, and GFR_max_ showed wide variations (Table 1). Grain size (GL, GW, and GT) had a significant correlation with grain weight (*P* < 0.001); and grain filling also showed a significant correlation with grain weight (*P* < 0.001; S2 Table). These results suggested that grain weight is not only determined by grain size but also by GFR. Grain weight showed closer relationship with grain size than grain filling.

### Identification of allelic variations in *TaCKX* associated with grain traits

**Fig 1:**
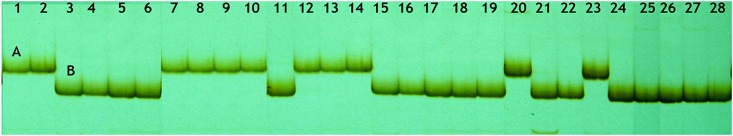
Allelic variation of *TaCKX6a02* in the RIL population of Jing 411/Hongmangchun 21. Lane Nos. (1) Jing 411; (2) JH1; (3) Hongmangchun 21; (4) JH19; (5) JH31; (6) JH38; (7) JH2; (8) JH3; (9) JH4; (10) JH5; (11) JH73; (12) JH9; (13) JH10; (14) JH12; (15) JH75; (16) JH79; (17) JH81; (18) JH103; (19) JH105; (20) JH13; (21) JH107; (22) JH113; (23) JH15; (24) JH118; (25) JH120; (26) JH125; (27) JH129; and (28) JH130. In the figure, JH1, JH19, JH31, JH38, etc., represent individual names of the RIL population. Two types of alleles, *TaCKX6a02-D1a* (Jing 411) and *TaCKX6a02-D1b* (Hongmangchun 21) are marked with the letters “A” and “B”, respectively.

Of the 24 primer pairs developed from wheat *CKX* genes (Galuszka et al. 2004)[31], six pairs including T3–4 (*TaCKX1*), T5–6 (*TaCKX2a*), T13–14 (*TaCKX3*), T19–20 (*TaCKX4*), T25–26 (*TaCKX5b*), and T31–32 (*TaCKX6a02*) showed good polymorphism between the two parents. To identify *TaCKX* genes associated with grain traits, spearman rank correlation analysis and t-test were performed based on the mean performance of grain traits of RILs in the five environments. The results indicated that the allelic variation of *TaCKX6a02* had a significant correlation with grain size, grain weight, and GFR in the RIL population (*P* < 0.001; Table 2). The alleles of the A- and B-type patterns shown in Fig 1 were named *TaCKX6a02-D1a* and *TaCKX6a02-D1b*, respectively. The marker used for identifying the allelic variation of *TaCKX6a02-D1* was designated as *CKX3D*. RILs carrying *TaCKX6a02-D1a* had significantly higher values for grain size, weight and grain filling than *TaCKX6a02-D1b* (Table 2), indicating that the *TaCKX6a02-D1a* allele could improve grain traits.

**Table 2:** Analysis of grain traits between two alleles of *TaCKX6a02*, and Spearman correlations and t-test between the allelic variation of *TaCKX6a02* and grain traits in the RILs and natural population.

Population	Alleles/Traits	GL/mm	GW/mm	GT/mm	TGW/g	GFRmax	GFRmean
RIL	*Tackx6a02-D1a*	6.89	3.34	3.20	41.91	2.94	2.04
	*Tackx6a02-D1b*	5.59	2.70	2.69	29.26	1.96	1.33
	t-test^a^	11.403***	9.668***	8.15***	14.45***	8.696***	7.863***
	Correlation^b^	0.585***	0.517***	0.414***	0.609***	0.471***	0.413***
Natural	*Tackx6a02-D1a*	6.55	3.22	3.23	41.6	3.27	2.19
	*Tackx6a02-D1b*	5.45	2.78	2.61	27.3	2.16	1.77
	t-test^a^	12.052***	8.464***	6.999***	8.304***	9.423***	6.712***
	Correlation^b^	0.608***	0.512***	0.383**	0.556***	0.489***	0.380**
	Effect^c^	35.1%	26.2%	13.1%	31.6%	22.7%	12.8%

^a^ *t*-test of the averages of grain traits between the two alleles of *Tackx6a02*.

^b^Correlation between allelic variation of *TaCKX6a02* and grain traits among 61 wheat varieties.

^c^Effect of allelic variation on the variability of grain traits, as estimated by *R* ^*2*^ based on the General Linear Model.

** indicates significant differences at *P* < 0.01.

*** indicates significant differences at *P* < 0.001.

### Chromosome location of *TaCKX6a02*

Previous studies indicated that wheat *CKX* genes belong to a large gene-family, distributed on several chromosomes, including 3A, 3B, 3D, 7A, and 7B; hence, it was necessary to locate *TaCKX6a02* before performing genetic linkage analysis in the RIL population. In this study, the chromosome location of *TaCKX6a02* was identified using Chinese Spring nulli–tetrasomics. The PCR products were amplified in N3AT3B, N3AT3D, N3BT3A, and N3BT3D, but were not present in N3DT3A or N3DT3B (S1 Fig), indicating that the *TaCKX6a02* gene is located on chromosome 3D.

### Linkage analysis of the *TaCKX6a02* gene and functional marker development

**Fig 2:**
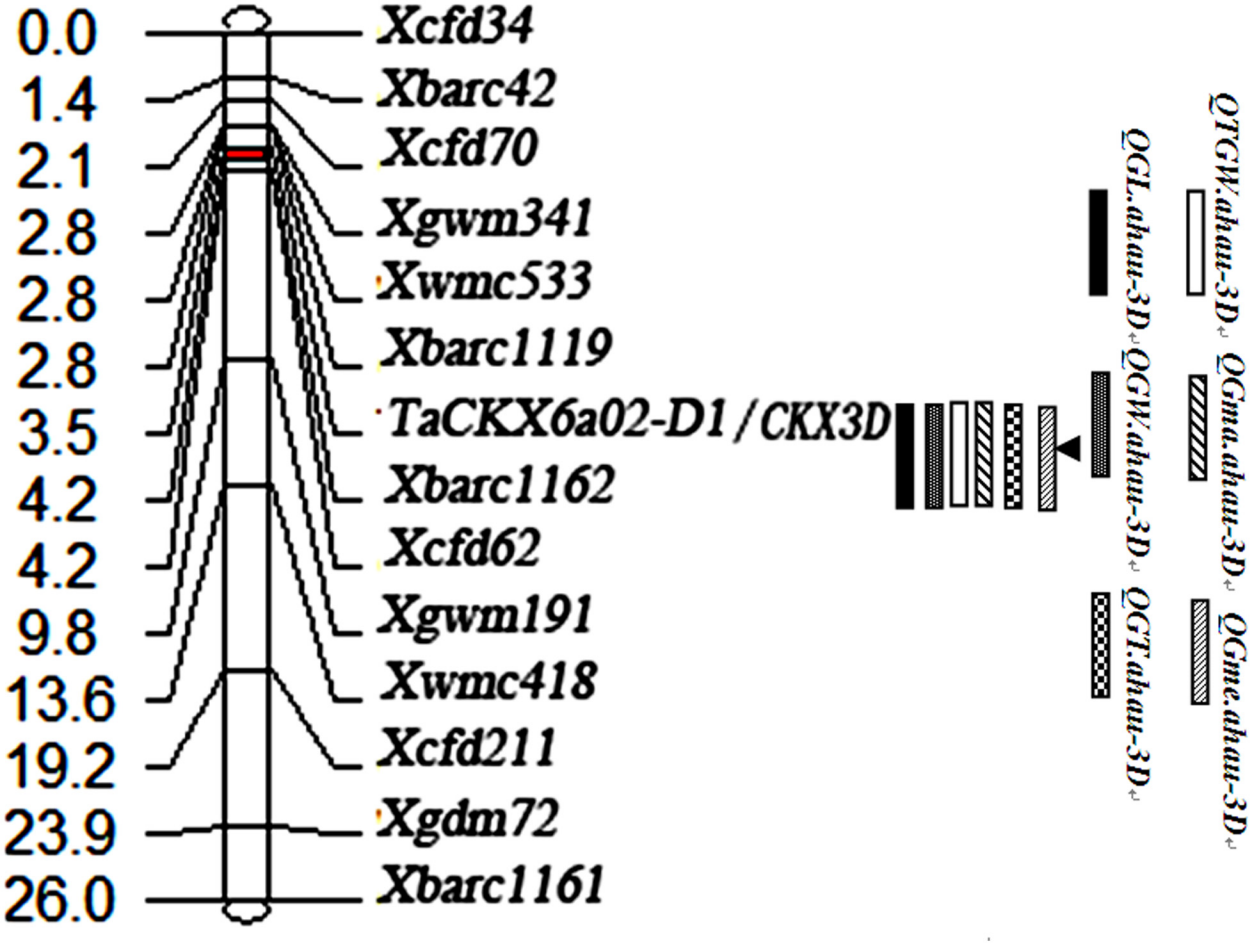
Linkage map of *TaCKX6a02* on chromosome 3DS of wheat. The six shaded boxes represent the six traits of TGW, GW, GL, GT, GFRmax, and GFRmean, respectively.

Seventy-seven SSR markers on chromosome 3D were selected based on their chromosomal location. To analyze the genetic linkage of *TaCKX6a02* on 3D, and to further evaluate its effect on the phenotypic variation of grain traits, the SSR markers were screened using their two parents and two bulks. Fourteen SSR markers and the gene-marker *CKX3D* showed good polymorphism in the mapping population. Genetic map analysis indicated that the 14 SSR markers were assigned in the same linkage group as the CKX3D region, spanning a genetic distance of 26 cM (Fig 2).

Through the analysis of CIM, a QTL controlling grain traits was identified in the marker interval of *Xbarc1119* and *Xbarc1162* on chromosome 3DS, with a genetic distance of 1.4 cM (Fig 2). The QTL co-segregated with *TaCKX6a02*, with LOD values ranging from 9.1 to 21.1 over five environments. The six grain traits, GL, GW, GT, TGW, GFR_max_, and GFR_mean_ were all associated with the same QTL closely linked to *TaCKX6a02*, *Xbarc1119*, and *Xbarc1162*.

The QTL mapped at *CKX3D* locus was detected across all five cropping seasons, and could explain 17.4~38.2% of phenotypic variations for grain size and grain weight (Table 3). Regarding grain filling traits, the locus also accounted for 17.1% and 22.2% of phenotypic variations of GFR_max_ and GFR_mean_, respectively, in the two environments (2009~2010 and 2010~2011 cropping seasons). Therefore, *TaCKX6a02* could be considered as a candidate gene for grain filling, grain size, and grain weight in this population.

**Table 3:** Summary of QTLs for grain traits in the RILs from the Jing 411/Hongmangchun 21 cross, across different environments.

Trait^a^	QTL	Marker interval	LOD	*R* ^*2*^ (%)	Add.(%)	Environments observed/total
GL	*QGL*.*ahau-3D*	*T31-32~Xbarc1162*	20.4	34.2	–0.6	5/5
GW	*QGW*.*ahau-3D*	*T31-32~Xbarc1162*	17.6	26.7	–0.76	5/5
GT	*QGT*.*ahau-3D*	*T31-32~Xbarc1162*	9.8	17.4	–11.2	5/5
TGW	*QTGW*.*ahau-3D*	*T31-32~Xbarc1162*	21.1	38.2	–1.18	5/5
GFR_max_	*QGma*.*ahau-3D*	*T31-32~Xbarc1162*	11.9	22.2	–0.49	2/2
GFR_mean_	*QGme*.*ahau-3D*	*T31-32~Xbarc1162*	9.1	17.1	–0.27	2/2

^a^All values are calculated from the overall mean dataset

### Validating effects of *TaCKX6a02* variations on grain traits among 102 wheat varieties

To further confirm effects of *TaCKX6a02* on grain traits, association analysis of allelic variations with grain traits were conducted among 102 wheat genotypes. In this natural population, 68.5% of varieties had the *TaCKX6a02-D1a* allele, while the remainder had *TaCKX6a02-D1b* (S3 table).

A wide variation of grain traits was observed among these wheat varieties (Table 1). The mean values of the six grain traits all showed significant differences (*P* < 0.001) between *TaCKX6a02-D1a* and *TaCKX6a02-D1b* (Table 2). The varieties with *TaCKX6a02-D1a* allele had significantly higher value of grain traits than those with *TaCKX6a02-D1b* allele. The association analysis showed that the allelic variation of *TaCKX6a02* had a significant correlation with grain traits in natural population of wheat (*P* < 0.01 or 0.001; Table 2; S2 Fig). Phenotypic variations of grain traits explained by *TaCKX6a02* ranged from 12.8~35.1% across the two cropping seasons (2011~2012, 2012~2013). Through analysis in different genetic background, RILs and natural population, *TaCKX6a02* could be validated to have a significant effect on grain traits in common wheat.

### Sequence analysis of *TaCKX6a02*

**Fig 3:**
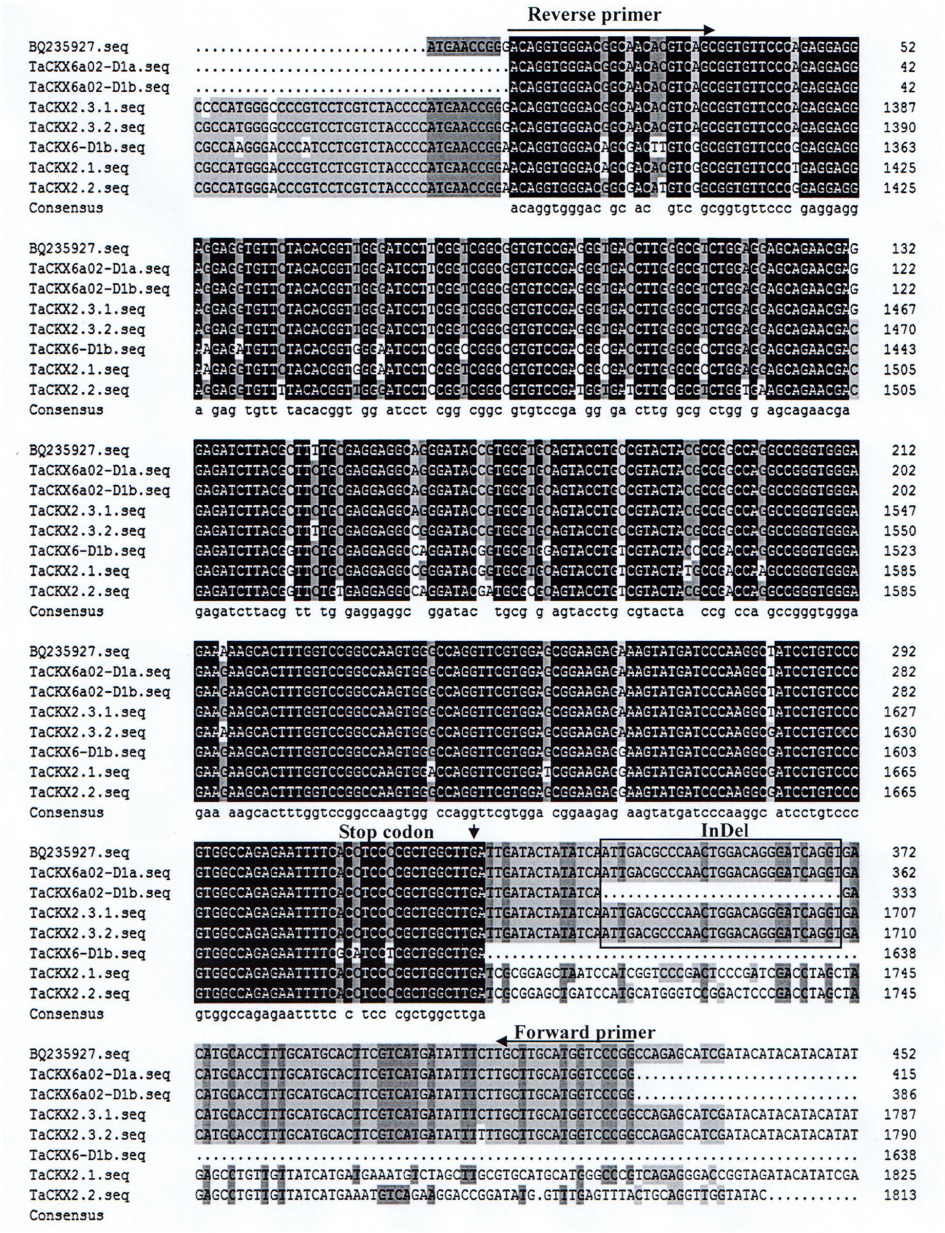
Sequence alignment between *TaCKX6a02* and *TaCKX2*.*1*, *TaCKX2*.*2*, *TaCKX2*.*3*.*1*, *TaCKX2*.*3*.*2*, and *TaCKX6-D1b*. Two alleles sequences of *Tackx6a02*, *TaCKX6a02-D1a*) and *TaCKX6a02-D1b* were obtained from the reverse-complement sequences of PCR fragments of Jing 411 and Hongmangchun 21. The primer pair and stop codon are marked with arrows in the figure. The InDel sequence is boxed.

A 29-bp insertion in the 3′-untranslation region (3′-UTR) behind the TGA stop codon was detected in the Jing 411 allele of *TaCKX6a02-D1a*, compared with the Hongmangchun 21 allele *TaCKX6a02-D1b* (Fig 3). The sequence of *TaCKX6a02-D1a* was the same as the EST BQ235927 deposited at the NCBI (Galuszka et al. 2004) [31]. Other *CKX* genes on chromosome 3D, including *TaCKX2*.*1* (FJ648070.1), *TaCKX2*.*2* (GU084177.1), *TaCKX2*.*3*.*1* (JF293079), *TaCKX2*.*3*.*2* (JN128584), and *TaCKX6-D1b* (JQ797673) were also obtained by performing a BLAST search on the NCBI GenBank. Sequences between *TaCKX6a02* and the above *TaCKX* genes were analyzed using DNAMAN software. The results revealed *TaCKX6a02* shared a high identity with them in the open reading frame region (ORF), as shown in Fig 3. Through alignment analysis among them, *TaCKX6a02* had higher homology with EST BQ235927 (99%), *TaCKX2*.*3*.*1* (99%) and *TaCKX2*.*3*.*2* (99%), than with *TaCKX2*.*1* (92%), *TaCKX2*.*2* (92%) and *TaCKX6-D1b* (92%) (S3 Fig). The *TaCKX*s on chromosome 3D could thus be divided into two groups according to their homology.

## Discussion

### Homology ananlysis of *CKX* genes on chromosome 3D of wheat

Six *CKX* genes on chromosome 3D of wheat were identified following a BLAST search in NCBI using the *TaCKX6a02* sequence, including an EST BQ235927, *TaCKX2*.*1*, *TaCKX2*.*2*, *TaCKX2*.*3*.*1*, *TaCKX2*.*3*.*2*, and *TaCKX6-D1b*. Alignment analysis revealed that *TaCKX6a02*, *TaCKX2*.*3*.*1* and *TaCKX2*.*3*.*2* are possibly the same *CKX* gene because of very high identity in sequence between them. However, the function of *TaCKX2*.*3*.*1* and *TaCKX2*.*3*.*2* had remained unknown until now. *TaCKX2*.*1*, *TaCKX2*.*2* and *TaCKX6-D1b* also shared higher sequence homology among them, but had a lower identity with *TaCKX6a02*. For example, *TaCKX6a02* had a large difference in sequence at the 3′-UTR, compared with *TaCKX2*.*1* and *TaCKX2*.*2*. The main variation in *TaCKX6a02*, 29-bp InDel in the 3′-UTR, was also different from *TaCKX6-D1b* containing 18-bp InDel in intron 2 and 20-bp InDel in the third exon [30]. According to these analyses, these *TaCKX* genes were clearly divided into two groups.

Multiple *CKX* genes have been observed on chromosome 3DS of wheat; however, their functions varied. *TaCKX2*.*1* and *TaCKX2*.*2* are involved in the regulation of grain number per spike in wheat [29], similar to *OsCKX2* in rice [27]. On the other hand, *TaCKX6* [30] and *TaCKX6a02* are associated with grain weight. Interestingly, these *CKX* genes have a relatively high sequence identity and all located on chromosome 3DS. Furthermore, *TaCKX6* and *TaCKX6a02* share a similar linkage interval on the genetic map. Based on sequence identity and linkage analysis, *TaCKX6* and *TaCKX6a02* may be different alleles of the *CKX* gene. Therefore, further study is to clone full length of *TaCKX6a02* and compare its sequence with *TaCKX6*.

### Allelic variation of *TaCKX6a02*

Gene mutations occurring in coding regions often affect function as a result of amino acid changes. However, the 5′-UTR [47, 48], 3′-UTR [49–52], and intron [30, 53] also have important influences on gene function through regulating mRNA structure, stability, and accumulation during translation. Previous research has confirmed that an 18-bp InDel in the second intron of *TaCKX6-D1b* had a significant influence on the gene expression at eight days after pollination in common wheat [30]. In this study, a novel 29-bp InDel was found in the 3′-UTR of *TaCKX6a02*. Whether variation of *TaCKX6a02* leads to enhancement of grain weight by directly increasing endogenous cytokinin levels, and what is the synergistic relationship between the two markers (C19L3⁄C19L4 and TKX3D) and grain weight are worthy of further study.

### *TaCKX6a02* locus for grain traits

As a complex trait, grain weight is controlled by multiple genes or loci widely distributing on the most of wheat chromosomes. On chromosome 3D, several QTLs for grain weight and related traits have been detected using varied populations, explaining 6~23% of phenotype variations in different environments [38, 54–58]. In this study, the *TaCKX6a02* locus for grain traits was closely linked to *Xgwm341*, *Xwmc533*, *Xbarc1119*, *Xbarc1162*, and *Xcfd62* on chromosome 3DS; the linkage intervals were similar to the previous reports [55, 56]. However, these loci explained relatively less phenotypic variation of grain traits, compared with the *TaCKX6a02* locus (17.1~38.2%). Zhang et al. [30] also reported that *TaCKX6* was located on chromosome 3DS and linked to *Xcfd70* and *Xwmc533*. This is very similar to *TaCKX6a02*. These results revealed that varied QTLs or loci for grain weight occurred on chromosome 3D of wheat.

Identification of genes for grain traits in wheat is receiving increased attention of researchers. At present, some genes for grain size and weight are known, including *TaGW2* [59–61], *TaCKX6* [30], *TaGS-D1* [62], *6-SFT-A2* [63]. In our study, *TaCKX6a02* is also confirmed to tightly relate to grain size and weight by QTL and association analysis. Interestingly, the gene also has significant effect on grain filling rate of wheat simultaneously. The result suggests that *TaCKX6a02* is likely involved both in regulating grain formation and the filling process of wheat. The influence of *TaCKX6a02* on grain weight was probably due to its regulation on both grain size and filling. Therefore, findings of variations in *TaCKX6a02* will help further understanding of complex mechanisms of grain weight. In addition, the *TaCKX6a02-D1a* is a predominant allele among modern varieties, and *TaCKX6a02-D1b* allele mainly distributes in local varieties or landraces of China in our study. These results show a strong selection of *TaCKX6a02-D1a* in breeding programs. Furthermore, the locus co-segregating with *TaCKX6a02* shows good stability and reliability in varied environments and genetic backgrounds, and thus will help improve the accuracy and effectiveness of marker assisted selection for grain traits in wheat breeding.

## Conclusions

*TaCKXs* belong to a big gene family, including at least 13 members in common wheat. Several *TaCKX* genes have been validated to relate to grain yield. In this research, *TaCKX6a02* on chromosome 3DS was also identified to closely associate with grain size, filling and weight in a RIL population and natural population respectively. By sequence analysis, a novel allelic variation of *TaCKX6a02* was observed, which contained a 29-bp InDel behind the stop codon. A gene-specific marker, *TKX3D*, was developed based on the InDel variation. The *TKX3D* locus could explain 12.8–38.2% of phenotypic variations in different environments.

## Supporting Information

S1 Table Primer pairs used in this study. (DOC)

S2 Table Correlation between the grain traits of RILs. (DOC)

S3 Table The information of grain weight, allele of *TaCKX6a02* and distributed region of 102 wheat varieties. (DOCX)

S1 Fig Amplification of the *TaCKX6a02* gene using the primer pair T31–32 in Chinese Spring nulli–tetrasomics. (TIF)

S2 Fig Grains of 12 wheat varieties with large difference in grain size and weight. (TIF)

S3 Fig Homology analysis of *TaCKX* genes. (TIF)
