# Supplementary material for: Identification of a Novel Allele of TaCKX6a02 Associated with Grain Size, Filling Rate and Weight of Common Wheat
Source: PLoS One. 2015 Dec 14;10(12):e0144765. doi: 10.1371/journal.pone.0144765 (PMC4685998; doi:10.1371/journal.pone.0144765)
Supplement: S1 Table — (DOC) [file pone.0144765.s001.doc]

S1 Table Primer pairs used in this study

| Primer | *CKX* gene^a^ | Forward sequence 5′-3′ | Reverse sequence 5′-3′ |
| --- | --- | --- | --- |
| T1-2 | *TaCKX1* | acgggtacatgctggagattaaag | aggtggcgctggacaagatc |
| T3-4 | *TaCKX1* | gcagcctcttggggtcgtact | actcgggcgggttcttcac |
| T5-6 | *TaCKX2a* | aggtgacatctttggttttctttg | cggagggcgaggtgttctac |
| T7-8 | *TaCKX2a* | ggcctccgtgcggtagtgc | cacccgtggctcaacctcttc |
| T9-10 | *TaCKX2b* | gctgctaatgcgcgatcttgc | atgcggcggctcaagtacgt |
| T11-12 | *TaCKX2b01* | ccctttccctacgacaacatcca | cacccgtggctcaacctcttc |
| T13-14 | *TaCKX3* | cacggcttgatgcatgcttcc | ccggagggcgaggtgttcta |
| T15-16 | *TaCKX3* | ccctataatcatcgtcatcgtcct | ccggagggcgaggtgttcta |
| T17-18 | *TaCKX4* | ccagttcggcatcatcaccag | tgtggaagagggttgactgtatgt |
| T19-20 | *TaCKX4* | aggttggtgtgctgctgtctc | ctccgctcaaatgtctcccac |
| T21-22 | *TaCKX4* | ggcgaggtgggagacatttga | tggtgggcttgtgtcgttactc |
| T23-24 | *TaCKX5a* | cggtgaagttggagtagagcg | cggggtggtggtggacat |
| T25-26 | *TaCKX5b* | tcggggccaggcagtacc | tgcgcgccacatacatgacac |
| T27-28 | *TaCKX5b01* | cgcgacgcattcctctgtacat | ccccatgaacaagcacaagtg |
| T29-30 | *TaCKX6a01* | gcctcctcgcagaatcgtaaga | ggttcgcgttcgtgcaggac |
| T31-32 | *TaCKX6a02* | ccgggaccatgcaagcaa | acaggtgggacggcaaca |
| T33-34 | *TaCKX6b* | cgggcagacacggggaac | gcaaagcgcgagaaatgacag |
| T35-36 | *TaCKX6b01* | gtgcggacgcttgccctc | agcgcgtgcgagagagatga |
| T37-38 | *TaCKX7a* | gggcgccagagaggtttatatg | ccggggtccaaggttcagg |
| T39-40 | *TaCKX7b* | gcctccaagaatcactcactcac | ggcccgtgcttgaatgtctg |
| T41-42 | *TaCKX7b* | gcggtgttgaagccccagtc | gggcgagcatgtcacggtct |
| T43-44 | *TaCKX8* | ggggctcatcctcatctatcc | agggcaacatacagatcgaacag |
| T45-46 | *TaCKX6b* | gcgcgagaaatgacagtgatag | cgggcagacacggggaac |
| T47-48 | *TaCKX6b01* | cgatcgttcacccaagctaag | gggcagacaccggggaac |

^a^*CKX* gene names were in accordance with the description given by Galuszka et al. [31].
