# Supplementary material for: Identification of a Novel Allele of TaCKX6a02 Associated with Grain Size, Filling Rate and Weight of Common Wheat
Source: PLoS One. 2015 Dec 14;10(12):e0144765. doi: 10.1371/journal.pone.0144765 (PMC4685998; doi:10.1371/journal.pone.0144765)
Supplement: S2 Table — (DOC) [file pone.0144765.s002.doc]

S2 Table Correlation between the grain traits of RILs

| Trait | GW | GT | TGW | GFR_max_ | GFR_mean_ |
| --- | --- | --- | --- | --- | --- |
| GL | 0.835*** | 0.697*** | 0.740*** | 0.337** | 0.323** |
| GW |  | 0.723*** | 0.607*** | 0.212* | 0.234* |
| GT |  |  | 0.568*** | 0.107^ns^ | 0.101 ^ns^ |
| TGW |  |  |  | 0.472*** | 0.415*** |
| GFR_max_ |  |  |  |  | 0.654*** |

Significant correlations at *P* levels of 0.05, 0.01, and 0.001 are marked with *, **, and ***, respectively; ns, not significant. Mean values are calculated from the overall dataset of five environments.
