# Supplementary material for: Identification of a Novel Allele of TaCKX6a02 Associated with Grain Size, Filling Rate and Weight of Common Wheat
Source: PLoS One. 2015 Dec 14;10(12):e0144765. doi: 10.1371/journal.pone.0144765 (PMC4685998; doi:10.1371/journal.pone.0144765)
Supplement: S3 Table — (DOCX) [file pone.0144765.s003.docx]

S3 Table. The information of grain weight, allele of *TaCKX6a02* and distributed region of 102 wheat varieties

| Variety name | Allele type | TGW/g | Origin | Variety name | Allele type | TGW/g | Origin |
| --- | --- | --- | --- | --- | --- | --- | --- |
| Nannong87-88 | *TaCKX6a02-D1a* | 40.7 | Jiangsu | Huaimai33 | *TaCKX6a02-D1a* | 43.7 | Jiangsu |
| Ningmaizi18 | *TaCKX6a02-D1a* | 37.5 | Jiangsu | Yannong19 | *TaCKX6a02-D1a* | 43.7 | Shandong |
| Yangmai5 | *TaCKX6a02-D1a* | 37.1 | Jiangsu | Chuanmai8 | *TaCKX6a02-D1b* | 36.2 | Sichuan |
| Yuguo | *TaCKX6a02-D1b* | 27.4 | Japan | Jimai34 | *TaCKX6a02-D1b* | 30.8 | Hebei |
| Zitongnv’ermai | *TaCKX6a02-D1b* | 20.3 | Sichuan | Jinan4 | *TaCKX6a02-D1b* | 38.7 | Shandong |
| Xiaoyuhua | *TaCKX6a02-D1b* | 23.7 | Jiangsu | Niavt14 | *TaCKX6a02-D1b* | 30.4 | - |
| Baimanghong | *TaCKX6a02-D1b* | 25.4 | Shanxi | Yumai2 | *TaCKX6a02-D1b* | 38.7 | Henan |
| Waibaitou | *TaCKX6a02-D1b* | 26.8 | Henan | ARZ | *TaCKX6a02-D1b* | 34.7 | - |
| Chadianhongmai | *TaCKX6a02-D1b* | 33.4 | Hebei | Wanmai38 | *TaCKX6a02-D1a* | 43.7 | Anhui |
| Baihuomai | *TaCKX6a02-D1b* | 28.9 | Shanxi | Zhoumai16 | *TaCKX6a02-D1a* | 46.7 | Henan |
| Huangguaxian | *TaCKX6a02-D1b* | 24.7 | Henan | Aikang58 | *TaCKX6a02-D1a* | 46.2 | Henan |
| Zhongmai 9 | *TaCKX6a02-D1a* | 52.1 | Beijing | Zhoumai18 | *TaCKX6a02-D1a* | 47.6 | Henan |
| Zhongmai11 | *TaCKX6a02-D1a* | 37.6 | Beijing | Xinmai18 | *TaCKX6a02-D1a* | 41.2 | Henan |
| Zhongmai18 | *TaCKX6a02-D1a* | 43.5 | Beijing | Mian39 | *TaCKX6a02-D1a* | 46.2 | Sichuan |
| Sanyuehuang | *TaCKX6a02-D1b* | 34.8 | Anhui | Shijiazhuang8 | *TaCKX6a02-D1a* | 45.9 | Hebei |
| Jinguangmai | *TaCKX6a02-D1b* | 27.8 | Anhui | Lankao298 | *TaCKX6a02-D1a* | 54.3 | Henan |
| Nongda45 | *TaCKX6a02-D1a* | 42.6 | Beijing | Fanmai5 | *TaCKX6a02-D1a* | 47.2 | Henan |
| Wenmai6 | *TaCKX6a02-D1b* | 35.7 | Henan | Kaimai18 | *TaCKX6a02-D1a* | 40.8 | Henan |
| Jimai20 | *TaCKX6a02-D1a* | 36.1 | Shandong | Jinhe9123 | *TaCKX6a02-D1a* | 41.8 | Henan |
| 955159 | *TaCKX6a02-D1a* | 47.8 | Shandong | Chuanmai42 | *TaCKX6a02-D1a* | 45.6 | Sichuan |
| Mian79-2 | *TaCKX6a02-D1b* | 37.8 | Sichuan | Annong1007 | *TaCKX6a02-D1a* | 45.9 | Anhui |
| Zheng9023 | *TaCKX6a02-D1a* | 57.1 | Henan | Liangxing66 | *TaCKX6a02-D1a* | 47.1 | Shandong |
| Funo | *TaCKX6a02-D1b* | 33.8 | Italy | Jimai22 | *TaCKX6a02-D1a* | 45.7 | Shandong |
| Shan225 | *TaCKX6a02-D1a* | 37.4 | Shanxi | Yunong69 | *TaCKX6a02-D1a* | 42.7 | Henan |
| Jimai14 | *TaCKX6a02-D1a* | 51.5 | Hebei | Xinmai19 | *TaCKX6a02-D1a* | 43.2 | Henan |
| Jimai9 | *TaCKX6a02-D1a* | 54.2 | Hebei | Yangmai20 | *TaCKX6a02-D1a* | 42.9 | Jiangsu |
| Nongda38 | *TaCKX6a02-D1a* | 50.4 | Beijing | Yanzhan4110 | *TaCKX6a02-D1a* | 47.2 | Henan |
| Jimai1 | *TaCKX6a02-D1a* | 46.3 | Hebei | Annong1124 | *TaCKX6a02-D1a* | 43.7 | Anhui |
| Yan’an11 | *TaCKX6a02-D1a* | 43.5 | Shanxi | Lunxuan988 | *TaCKX6a02-D1a* | 46.9 | Beijing |
| Shan160 | *TaCKX6a02-D1a* | 31.3 | Shanxi | Zhongmai895 | *TaCKX6a02-D1a* | 47.2 | Beijing |
| Bainong64 | *TaCKX6a02-D1a* | 41.2 | Henan | Annong0942 | *TaCKX6a02-D1a* | 49.2 | Anhui |
| Yumai18 | *TaCKX6a02-D1a* | 40.1 | Henan | Hengguan35 | *TaCKX6a02-D1a* | 40.1 | Hebei |
| Yangmai158 | *TaCKX6a02-D1a* | 41.7 | Jiangsu | Yannong21 | *TaCKX6a02-D1a* | 42.3 | Shandong |
| Gaiyuerui | *TaCKX6a02-D1b* | 37.6 | Xinjiang | Yangmai18 | *TaCKX6a02-D1a* | 38.7 | Jiangsu |
| 9114 | *TaCKX6a02-D1b* | 35.6 | Xinjiang | Zhoumai28 | *TaCKX6a02-D1a* | 41.2 | Henan |
| Tachun3 | *TaCKX6a02-D1b* | 38.4 | Xinjiang | Luo6073 | *TaCKX6a02-D1a* | 40.7 | Henan |
| Tachun2 | *TaCKX6a02-D1b* | 34.5 | Xinjiang | Huaimai0705 | *TaCKX6a02-D1a* | 46.9 | Jiangsu |
| 8612 | *TaCKX6a02-D1b* | 37.8 | Xinjiang | Yangmai08-4 | *TaCKX6a02-D1a* | 38.3 | Jiangsu |
| Neixiang203 | *TaCKX6a02-D1b* | 40.4 | Henan | Xuke129 | *TaCKX6a02-D1a* | 43.8 | Henan |
| Neixiangbodjiang | *TaCKX6a02-D1b* | 35.2 | Henan | Yangnuomai1 | *TaCKX6a02-D1a* | 38.2 | Jiangsu |
| ZM2855 | *TaCKX6a02-D1b* | 33.7 | Xinjiang | Yangmai20 | *TaCKX6a02-D1a* | 40.3 | Jiangsu |
| ZM2851 | *TaCKX6a02-D1b* | 38.6 | Xinjiang | Qianmai18 | *TaCKX6a02-D1a* | 37.2 | Guizhou |
| Hongmangchun21 | *TaCKX6a02-D1b* | 19.7 | Neimenggu | Qing18 | *TaCKX6a02-D1a* | 40.3 | Shanxi |
| Hongmangchun31 | *TaCKX6a02-D1b* | 25.2 | Neimenggu | Henong825 | *TaCKX6a02-D1a* | 42.4 | Henan |
| Jing411 | *TaCKX6a02-D1a* | 47.6 | Beijing | Zhengmai883 | *TaCKX6a02-D1a* | 45.2 | Henan |
| Yumai8679 | *TaCKX6a02-D1a* | 61.2 | Henan | Longke0901 | *TaCKX6a02-D1a* | 43.7 | Anhui |
| Zhongyou9507 | *TaCKX6a02-D1a* | 50.1 | Beijing | Huaihe0308 | *TaCKX6a02-D1a* | 45.4 | Jiangsu |
| Heshangmai | *TaCKX6a02-D1b* | 31.2 | Shanxi | Yi00-119 | *TaCKX6a02-D1a* | 43.4 | Sichuan |
| Chinese Spring | *TaCKX6a02-D1b* | 30.1 | Sichuan | 02p67 | *TaCKX6a02-D1a* | 41.5 | Jiangsu |
| Annong0711 | *TaCKX6a02-D1a* | 40.9 | Anhui | Wanmai52 | *TaCKX6a02-D1a* | 43.9 | Anhui |
| Shimai12 | *TaCKX6a02-D1a* | 47.1 | Hebei | Zhoumai22 | *TaCKX6a02-D1a* | 46.5 | Henan |

– data could not be determined
