# Supplementary figures and images for: Identification of a Novel Allele of TaCKX6a02 Associated with Grain Size, Filling Rate and Weight of Common Wheat
Source: PLoS One. 2015 Dec 14;10(12):e0144765. doi: 10.1371/journal.pone.0144765 (PMC4685998; doi:10.1371/journal.pone.0144765)

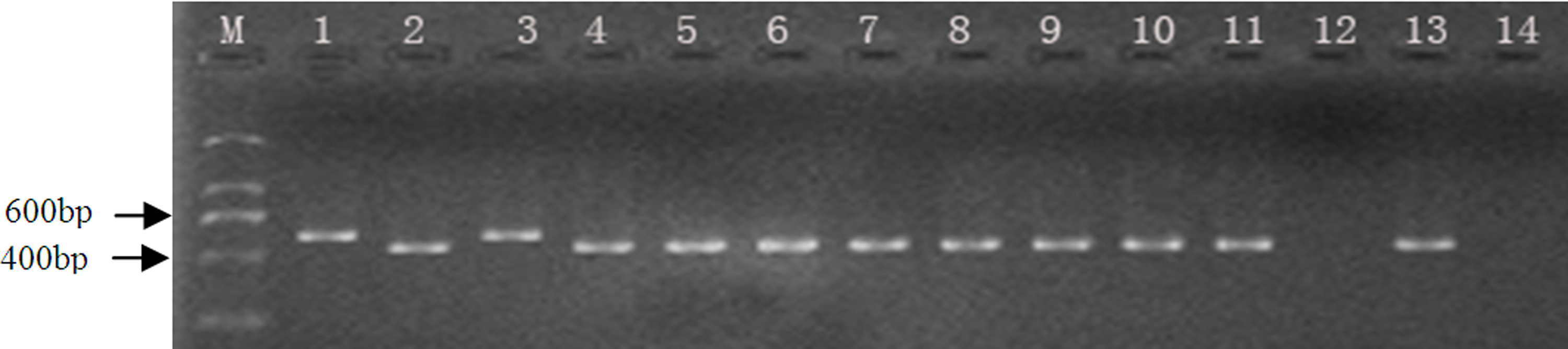

Supplement: S1 Fig — (TIF) [file pone.0144765.s004.tif]

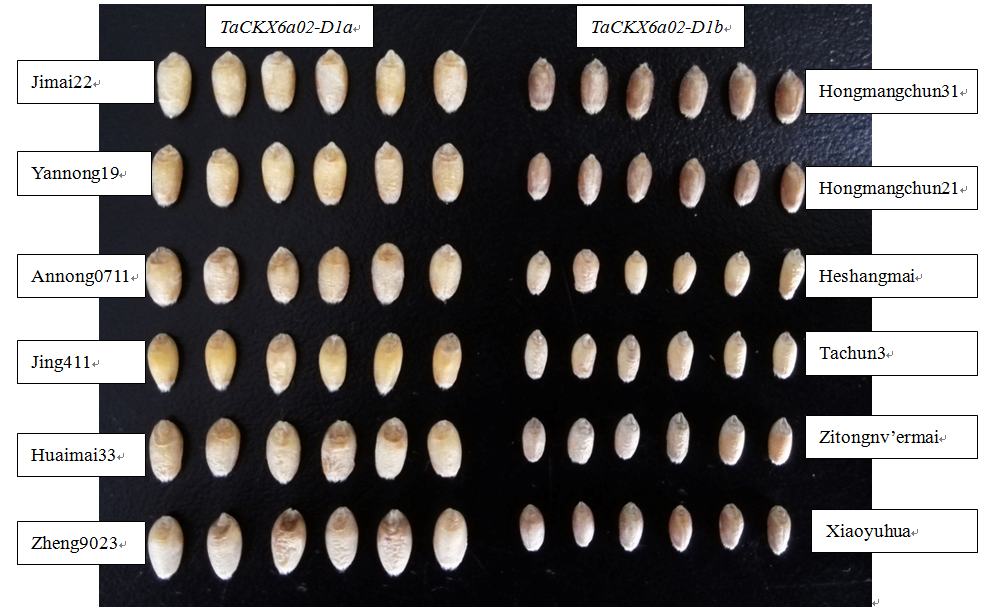

Supplement: S2 Fig — (TIF) [file pone.0144765.s005.tif]

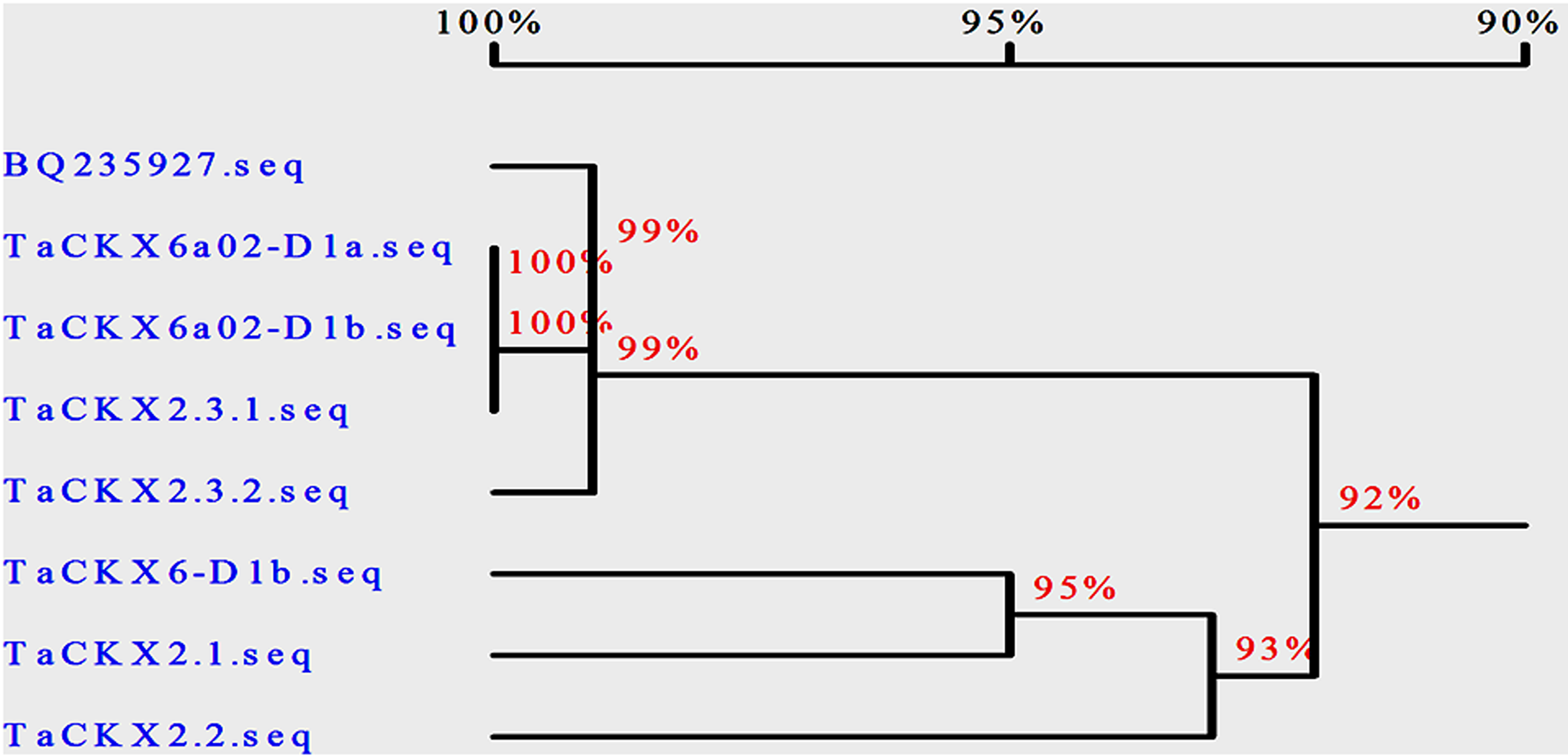

Supplement: S3 Fig — (TIF) [file pone.0144765.s006.tif]
